# Bacterial Contamination of Inhalation Chambers Used for Cats and Dogs with Chronic Airway Diseases

**DOI:** 10.3390/pathogens12020275

**Published:** 2023-02-08

**Authors:** Friederike Karoline Klenk, Vanessa De Simoi, Yury Zablotski, Bianca Désirée Ballhausen, Georg Wolf, Bianka Schulz

**Affiliations:** 1 Clinic of Small Animal Medicine, Centre for Clinical Veterinary Medicine, Ludwig Maximilians University of Munich, 80539 Munich, Germany; 2 Anicura Small Animal Clinic Haar, 85540 Haar, Germany; 3 Institute for Infectious Diseases and Zoonoses, Ludwig Maximilians University of Munich, 80539 Munich, Germany

**Keywords:** inhalation therapy, asthma, spacer devices, cleaning methods

## Abstract

Inhalation chambers (ICs) are regularly used in veterinary medicine for the inhalative treatment of chronic respiratory diseases in dogs and cats. Since therapy is usually required lifelong and daily, devices are frequently in use. The aim of this study was to identify bacterial contamination of ICs used for cats and dogs in relation to the applied cleaning measures. Swabs from ICs of 66 cats and 19 dogs with chronic airway diseases were obtained using a standardized protocol and subsequently cultured. A questionnaire was completed by the pet owners regarding the history of their pet’s illness and applied device cleaning measures. Overall, 64% (54/86) of the ICs were found to be contaminated; the mask was significantly (*p* < 0.001) more often contaminated than other device parts. Most cultured bacteria were environmental contaminants; however, some harbored pathogenic potential. Cleaning frequency and method did not significantly influence the presence of contamination. Bacterial contamination of ICs, used for cats and dogs, is common but is not significantly influenced by the type or frequency of cleaning. To avoid potential infection by opportunistic bacteria, the instruction of pet owners regarding the maintenance of the ICs is recommended.

## 1. Introduction

Chronic diseases of the lower airways are common in dogs and cats [1,2,3]. Feline asthma and feline chronic bronchitis are thought to affect at least 1% of the feline population [4,5]. In dogs, chronic bronchitis is considered one of the most common chronic respiratory diseases [3,6]. Especially in younger dogs, canine eosinophilic bronchopneumopathy represents a common chronic inflammatory respiratory condition [7].

Even though the etiology and pathophysiology of these chronic airway diseases are thought to be different, long-term therapy for all the above-mentioned conditions is similar, since the mainstay of drug therapy is glucocorticoids [8,9,10]. In dogs and cats, these were commonly administered orally or by injection [1,11].

In human medicine, the treatment of chronic respiratory diseases such as bronchial asthma and chronic bronchitis has been intensively investigated, and effective treatment strategies have been developed [12,13]. Inhaled therapy has been successfully used in human medicine for decades and is suggested to be the gold-standard treatment for patients with asthma or other chronic obstructive airway diseases [14,15]. Due to remaining concerns about possible side effects associated with long-term glucocorticoid therapy and coordination difficulties, especially in infants and children, improvement in aerosol delivery has been pursued through the development of inhalation aids such as spacers and valved holding chambers (VHC), hereafter referred to as inhalation chambers (ICs) [16,17,18]. The IC is used as an accessory device to pressurized metered dose inhalers, which contain aerosolized drugs such as glucocorticoids or bronchodilators [19,20]. The additional space between the metered dose inhaler and the patient, through the IC, reduces the particle size and velocity of the aerosol and eliminates the need to coordinate the spray actuation and inspiration [21,22,23]. The reduction in particle size results in more therapeutically effective small particles being able to reach deep into the airway and larger particles remaining in the ICs instead of being deposited in the oropharynx, thereby reducing local side effects [14,21,24]. Through the use of ICs, originally intended for use by children and infants, the inhalative application of corticosteroids is also possible for animals [11,25,26]. For this purpose, ICs, in combination with facemasks, have been developed for veterinary use [27,28]. Targeting drugs directly to the airways instead of systemic application aims to reduce side effects arising from long-term glucocorticoid administration while preserving local therapeutic effects [2,29,30].

Since ICs are permanently in use, come in contact with mucous membranes, and are usually not kept in a sterile environment, the question about possible bacterial contamination and the necessity of preventive cleaning measures arises [24,31]. Manufacturers of ICs recommend regularly cleaning the devices and replacing them after one year of use [32,33]. However, to date, it has not been investigated whether bacterial contamination is common in devices used in veterinary medicine, and if cleaning measures might have an impact on contamination.

Therefore, the objective of this study was to evaluate the degree of bacterial contamination in ICs used for cats and dogs in relation to the cleaning measures applied by the owners.

## 2. Materials and Methods

### 2.1. Study Design and Animals

In this prospective study, 85 ICs of client-owned cats (*n* = 66) and dogs (*n* = 19) were sampled. ICs of two different manufacturers that are commonly available in Germany were evaluated for bacterial contamination. For cats, devices included AeroKat^®^ (*n* = 53) (Trudell Medical International, London, ON, Canada) and RC Animal Chamber^®^ (*n* = 12) (Cegla Medizintechnik GmbH & Co. KG, Montabaur, Germany). For dogs, AeroDawg^®^ (*n* = 12) (Trudell Medical International, London, ON, Canada) and RC Animal Chamber^®^ (*n* = 7) (Cegla Medizintechnik GmbH & Co. KG, Montabaur, Germany) were sampled.

Inclusion criteria were regular use of one of the above-mentioned spacer devices over a period of at least one month before sampling.

### 2.2. Sample Collection

Three samples were collected from each IC according to a standardized protocol. For this purpose, sterile swabs with nutrient medium (Amies Transportmedium^®^, Sarstedt, Nuebrecht, Germany) were used. The samples were taken from the spacer itself, making two rotations through the inside of the chamber with one swab. The second sample was taken with another swab from the valve level, performing one rotation from the chamber side, the other from the mask side. The third sample was taken with a swab from the mask, making two rotations through the inside of the facemask.

All samples were collected by previously instructed personnel; in 83/85 cases, these were veterinarians. Only two AeroKat ICs were sampled by the cat owners themselves, who had received detailed instructions about the sampling protocol in advance.

Every sample was sent to the Department of Bacteriology and Mycology of the Institute for Infectious Diseases and Zoonoses of LMU University of Munich for cultivation within 48 h after sampling.

### 2.3. Bacteriological Examination

The samples were applied to culture media using standard techniques. Culture media included Bordetella Agar (Difco^TM^ Bordet Gengou Agar Ref#248200, Becton Dickinson, Le Pont de Claix, France (BD) with 15% sheep blood), BBL^TM^ Columbia Agar with 5% sheep blood (Ref# 211124, BD) and BBL^TM^ Columbia colistin nalidixic acid (CNA) agar with 5% sheep blood (Ref 212104, BD) in order to provide appropriate media for a variety of aerobic bacterial species.

The inoculated agar plates were incubated at 36–38 °C and examined for bacterial growth after 24, 48, and 72 h. Bacterial growth was semiquantitatively assessed. Degree of contamination was defined as negligible (<10 colony forming units (CFU)), moderate (10–50 CFU), or severe (>50 CFU). If bacterial growth was identified, bacterial species were further classified using matrix-assisted laser desorption time of flight mass spectrometry (MALDI-TOF, Microflex LT and MALDI Biotyper Identification-Software 3.1, Bruker Daltonik GmbH, Bremen, Germany; Library: Bruker Taxonomy Tree (8599 Spectra)).

### 2.4. Owner Questionnaire

At the time of sampling, owners completed a questionnaire about the use and maintenance of their pet’s inhalation device. The questionnaire consisted of three sections. The first section included general questions about the IC, including product type, duration of use, potential replacement of the IC, and drug formulation used with the device.

In the second section, owners had to answer questions about the animal’s airway disease, including potential infections that occurred under inhalation therapy.

In the third section, owners had to specify their cleaning routine, including method and frequency of cleaning and drying the device.

### 2.5. Statistical Analysis

Data were collected and analyzed with Microsoft Excel (V16.65). For statistical analysis of contaminated device parts, association between duration of use, and presence of contamination, as well as association between cleaning frequency and presence of contamination, the chi-square test was used. Fisher’s exact test was performed using SPSS (IBM SPSS Statistics, V 28.0.1.1 (14)) and was applied for nominal data with sample size of less than 5. Therefore, the test was used to evaluate if a relationship between cleaning method and presence of contamination was present and for assessment of an association between cleaning frequency and degree of contamination. Significance level was set at *p* < 0.05 for all comparisons. Graphs were plotted using Microsoft PowerPoint (V16.65).

## 3. Results

### 3.1. Sample Population

Samples were taken from devices used in 66 cats and 19 dogs. Feline asthma was the most common diagnosis in cats (57/66; 86.4%), followed by feline chronic bronchitis (7/66; 10.6%). Other indications for inhalation therapy included chronic rhinitis and not further investigated chronic cough (one each; 3.0%). In dogs, eosinophilic bronchopneumopathy was the most common disease (10/19; 52.6%), followed by canine chronic bronchitis (5/19; 26.3%). Other diseases (4/19; 21.0%) were bronchiectasis, tracheal collapse, and not further investigated chronic cough.

### 3.2. Presence of Contamination

In total, 54 of 85 sampled devices were found to be contaminated (64.0%). In the canine samples, 16/19 (84.2%) devices, and in the feline samples, 38/66 (57.6%) devices were contaminated, respectively. Among the contaminated devices, 30/54 (55.5%) showed negligible, 18/54 (33.3%) moderate, and 6/54 (11.1%) severe bacterial growth. Most prone to contamination was the mask, which was contaminated in 46/54 (85.2%) contaminated devices, followed by the chamber in 23/54 (42.6%) and the adapter in 20/54 (37.0%) cases. The mask was significantly more frequently contaminated than the adapter (*p* < 0.001) or the chamber (*p* < 0.001).

### 3.3. Isolated Microorganisms

Bacteria are displayed in Table 1 according to their taxonomic families and species for a better overview. In most samples, multiple microbial species could be identified. *Staphylococcus* spp. were the predominant bacteria, followed by *Acinetobacter* spp., *Micrococcus* spp., and *Bacillus* spp.

### 3.4. Device Use and Maintenance

Pet owners specified their cleaning frequencies as daily, after every use respectively (12/85; 14.1%), weekly (35/85; 41.2%), monthly (16/85; 18.8%), or less common/never (22/85; 25.8%). There was no association between the frequency of cleaning and contamination of the device (Table 2).

Furthermore, the degree of contamination did not significantly differ with regard to the frequency of cleaning (*p* = 0.06); results are shown in Table 3.

Regarding the duration of use of the IC, owners reported that 19 devices had been in use for less than six months (22.3%), 20 between six months and one year (23.5%), and 46 for more than one year (54.1%). There was no statistically significant association between the presence of contamination and the duration of use of the device (*p* = 0.99).

**Figure 1:**
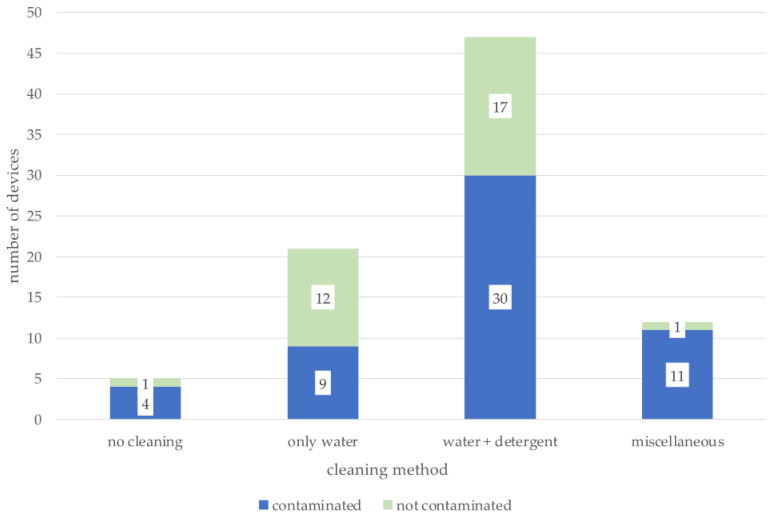
Presence of contamination, depending on cleaning method. Number of contaminated and clean devices in green and blue is shown for each cleaning method. Miscellaneous summarizes individual cleaning methods. Contamination did not significantly differ among the evaluated cleaning methods (*p* = 0.07).

Finally, the cleaning method was evaluated in context with the presence of contamination; results are shown in Figure 1.

Most owners stated that they cleaned with water and dishwashing detergent (47/85; 55.3%), followed by water only (21/85; 24.7%), miscellaneous methods (12/85; 14.1%), and owners who reported never cleaning their pet’s device (5/85; 5.9%). The applied cleaning method had no significant impact on the presence of contamination in the samples (*p* = 0.07).

## 4. Discussion

To the authors’ knowledge, this is the first study evaluating bacterial contamination of inhalation devices in veterinary medicine so far.

In the present study, 64% of the sampled devices were contaminated. The contamination rate of the masks was significantly higher than the contamination of the adapter and chamber. Since the mask comes in contact with the patient’s muzzle and the surrounding environment, a higher contamination of this part was expected. In the case reports of two cats and a dog, local demodicosis of the skin area, which came in contact with a facemask, occurred after therapy with inhalative glucocorticoids, presumably due to local immunosuppression [34,35]. In addition, it seems possible that contact with a mask contaminated with potentially pathogenic bacteria might also be a source of skin or wound infections. Since masks are usually made of silicon and do not have antistatic coating as some chambers do, more intensive cleaning or even disinfection of this part could be a possibility to avoid local infection induced by local immunosuppressive effects of the inhalative glucocorticoids.

Most bacterial species isolated in this study account as commensals of the skin or bacteria commonly found in the environment; therefore, their isolation from the IC is not surprising. Human and pet skin come in contact with the device during use, as well as the nose and muzzle of the patient, which are covered by the mask. The most common contaminants were *Staphylococcus* (*S*.) spp., isolated from 50 samples; among them, *S. hominis* was most frequently detected, followed by *S. epidermidis* and *S. pseudintermedius*. *S. hominis* and *S. epidermidis* are part of the resident skin flora in humans [36]. *Staphylococci*, especially *S. epidermidis*, has also been recognized as a main component of the canine and feline nasal and skin microbiota [37,38,39]. *S. pseudintermedius,* which was isolated from five devices, is a commensal of the skin, but is also considered potentially pathogenic, causing otitis externa and skin or urinary tract infections in susceptible dogs and cats [40,41]. *Micrococcaceae* were the second most common bacteria isolated, closely followed by *Moraxcellaceae* in this study. *Micrococcus* spp. are commonly isolated from the skin of dogs and cats. Among the *Moraxcellaceae*, *Moraxella* spp. belong to the predominant commensal species of the oral and nasal cavity in cats and dogs; *Acinetobacter* spp. are also described as a regular component of the canine nasal and skin microbiota [37,38,39,42,43]. *Bordetella* species could not be identified in any of the samples. *Bordetella bronchiseptica* is considered a primary pathogen of the respiratory tract in cats and dogs, involved in various respiratory tract diseases [44,45]. The duration of environmental persistence has not been conclusively investigated for this pathogen, but is thought to last at least 10 days [44]. Furthermore, *Pseudomonas* spp. were detected in seven samples. This organism can be found ubiquitous in the environment as well as in low numbers on the skin and in the airways of healthy animals [37,38,46,47]. However, *Pseudomonas* spp. are also considered one of the main species involved in cats with rhinitis [48].

The presence of microbial contamination in inhalation equipment has especially been studied in human cystic fibrosis patients, since respiratory infections are the primary cause of death in these patients [49,50,51,52]. In addition, there are some studies that evaluated the bacterial contamination of nebulizers and spacers used by human patients with asthma. Cohen and coworkers found 35.5% of the spacers used by asthmatic children to be contaminated. Some of the isolated bacteria were considered potentially pathogenic, including *Pseudomonas aeruginosa*, *S. aureus*, and *Klebsiella pneumoniae*. In contrast to our study, in that investigation, device samples with less than 10 colony-forming units were defined as clean [53]. In a similar study evaluating the contamination of nebulizers used by asthmatic children, 66.7% of the nebulizers were found to be contaminated with microorganisms, predominantly *Pseudomonas aeruginosa* and *Klebsiella pneumoniae* [54]. Lower numbers of 38% contaminated spacer devices were detected in another study evaluating the contamination of spacer devices used by children with asthma. However, only device samples with over 50 colony-forming units were defined as contaminated. In that study, predominantly environmental bacteria, most often *Bacillus* spp. were detected [55]. Most isolated bacteria in the present study are considered environmental inhabitants or a regular part of the nasal, oral, or cutaneous microbiome in dogs and cats. This leads to the assumption that the bacteria originated from the patient itself or its direct surroundings. Nevertheless, overexposure to potentially pathogenic bacteria might affect the patient’s health, especially for chronically ill patients. It is known that dysbiosis of the microbiome can lead to an increase in opportunistic pathogenic bacteria, leading to secondary infections [48]. Secondary bacterial infections might promote exacerbations of chronic respiratory diseases due to increased respiratory stress [56]. If reinfection through contaminated inhalation equipment is possible, and if the use of contaminated equipment promotes disease, exacerbations cannot be proven with certainty so far. In human medicine, studies have been performed to evaluate if contamination of inhalation devices is linked to insufficient asthma control. It could be shown that contamination of the devices and frequency of cleaning had no impact on the course of the disease [57,58]. In the present study, disease control had not been investigated, but would be an interesting aspect to include in future studies.

In the present study, the cleaning frequency did not significantly influence the presence or degree of bacterial contamination. ICs were regarded as contaminated, even if only low numbers of <10 CFU were detected. Samples with 10–50 CFU were defined as moderately contaminated, and those with >50 CFU as severely contaminated. In a veterinary study evaluating the contamination of breathing systems used during anesthesia, only very low numbers of bacteria, <10 CFU per sampling site, could be found, and the amount did not increase over the two-month study period [59]. A human study reported similar results in anesthesia systems, with an average of 1–9 CFU detected at every sampling site [60]. Even though breathing systems used in anesthesia differ greatly from the ICs studied here, we chose to take even those ICs with negligible, very mild contamination (<10 CFU) into account as contaminated. Since this study is the first in the veterinary field to examine the bacterial contamination of inhalation chambers, we chose this classification of the degree of contamination to provide a comprehensive overview of the results.

In the questionnaire used in this study, only 5.1% of the pet owners reported signs of a bacterial respiratory tract infection during inhalation therapy. However, it should be noted that most owners could not differentiate between respiratory signs due to a secondary infection of the airways and their pets’ chronic respiratory signs because of the underlying disease; therefore, secondary bacterial infections might have been underdiagnosed in some cases. In addition, owners who reported a secondary bacterial infection during inhalation therapy might have mistaken the signs of disease exacerbation for a secondary infection.

The duration of IC use had no impact on the presence of contamination either; however, manufacturers of spacer devices suggest annual replacement of the chambers. Looking at the results of this study, replacement does not seem necessary to avoid contamination, but might be advisable to maintain sufficient IC function.

Proper drying of nebulizers and spacers resulted in lower contamination rates in human studies, presumably because Gram-negative bacteria, frequently colonizing the environment, less commonly survive in dry environments [52,53,54]. Insufficient drying of the ICs might be an explanation for a slightly higher contamination rate, regarding absolute numbers, observed in the chambers that were cleaned daily or after every use (66.7%) compared with those only cleaned once a week (58.8%). Another explanation would be more frequent use of the more frequently cleaned devices resulting in greater exposure to environmental bacteria [55].

The limitations of this study were a relatively small sample size and the unknown reliability of the clients’ answers in the questionnaire. In addition, even if the clients were asked not to change their cleaning habits before sampling, it cannot be excluded that some might have cleaned differently or more intensively prior to sampling. Furthermore, defining ICs with very low numbers of isolated bacteria (<10 CFU) as contaminated might have led to overreporting of bacterial contamination. It must be taken into account that such minor bacterial growth could also be due to contamination during the sample collection and handling process and, therefore, might not represent the actual degree of contamination appropriately. Further studies that build on the results of this study could use duplicate or triplicate sampling protocols to exclude accidental contamination occurring in the sample handling process and narrow down the actual amount of contamination and type of bacteria more precisely.

## 5. Conclusions

Although ICs used for cats and dogs are commonly contaminated with bacteria, most are predominantly commensals of the regular cutaneous, nasal, and oral microbiome. The role of contaminated spacers in disease control and prevention of exacerbations currently remains unclear. Nevertheless, proper spacer maintenance and cleaning are advised, not only to reduce the risk of exposure to potential pathogens, but also to maintain adequate spacer function.

Only 28% of pet owners cleaned their devices according to manufacturers’ instructions. Clients should be instructed by their veterinarians about the proper use and maintenance of the device to ensure optimal drug delivery and patient safety.

## Figures and Tables

**Table 1:** Isolated bacteria.

Family	Genus	Number (%) of Isolates
*Staphylococcaceae*	*Staphylococcus* spp.	50 (30.9)
*Moraxellaceae*	*Acinetobacter* spp.	19 (11.8)
	*Moraxella* spp.	4 (2.5)
*Micrococcaceae*	*Micrococcus* spp.	14 (8.6)
	*Rothia* spp.	3 (1.8)
	*Kocuria* spp.	4 (2.5)
	*Pseudarthrobacter* spp.	3 (1.8)
*Bacillaceae*	*Bacillus* spp.	14 (8.6)
	*Alkalihalobacillus* spp.	1 (0.6)
	*Lysinibacillus* spp.	1 (0.6)
	*Priestria* spp.	1 (0.6)
*Pseudomonadaceae*	*Pseudomonas* spp.	7 (4.3)
*Microbacteriaceae*	*Pseudoclavibacter* spp.	1 (0.6)
	*Microbacterium* spp	1 (0.6)
*Caulobacteriaceae*	*Brevundimonas* spp.	2 (1.2)
*Enterobacteriaceae*	*Enterobacter* spp.	1 (0.6)
	*Leclercia* spp.	1 (0.6)
*Enterococcaceae*	*Enterococcus* spp.	2 (1.2)
Others		16 (9.9)
Bacteria without further differentiation		14 (8.6)
Fungi/hyphae		3 (1.8)

**Table 2:** Contamination of inhalation chambers by frequency of cleaning (*n* = 85).

Presence of Contamination				
Cleaning Frequency	Contaminated	Clean	Total Number	*p*-Value
daily/after every use	8	4	12	0.72
weekly	20	14	34	
monthly	10	7	17	
less common/never	16	6	22	

**Table 3:** Degree of contamination in inhalation chambers by frequency of cleaning (*n* = 85).

Degree of Contamination					
Cleaning Frequency	Not Contaminated	Negligible	Moderate	Severe	*p*-Value
daily/after every use	4	2	3	3	0.06
weekly	14	16	4	0	
monthly	7	3	5	2	
less common/never	6	9	6	1	

## Data Availability

The authors confirm that all data analyzed in the study are available from the corresponding author upon reasonable request.
